# Influence of Area, Age and Sex on Per- and Polyfluorinated Alkyl Substances Detected in Roe Deer Muscle and Liver from Selected Areas of Northern Italy

**DOI:** 10.3390/ani14040529

**Published:** 2024-02-06

**Authors:** Susanna Draghi, Giulio Curone, Radmila Pavlovic, Federica Di Cesare, Petra Cagnardi, Claudia Fornesi Silva, Alberto Pellegrini, Federica Riva, Francesco Arioli, Marco Fidani

**Affiliations:** 1 Department of Veterinary Medicine and Animal Sciences, University of Milan, Via dell’Università 6, 26900 Lodi, Italy; susanna.draghi@unimi.it (S.D.); giulio.curone@unimi.it (G.C.); petra.cagnardi@unimi.it (P.C.); federica.riva@unimi.it (F.R.); francesco.arioli@unimi.it (F.A.); 2 Proteomics and Metabolomics Facility (ProMeFa), IRCCS San Raffaele Scientific Institute, Via Olgettina 60, 20132 Milan, Italy; pavlovic.radmila@hsr.it; 3 UNIRELAB S.r.l., Via Gramsci 70, 20019 Settimo Milanese, Italy; c.fornesi@unirelab.it (C.F.S.); a.pellegrini@unirelab.it (A.P.); m.fidani@unirelab.it (M.F.)

**Keywords:** biomonitoring, wild animals, ecotoxicology, high-resolution mass spectrometry, endocrine disruptors, PFAS

## Abstract

**Simple Summary:**

Per- and polyfluorinated alkyl substances (PFASs) bioaccumulate in living organisms with adverse health effects. Biomonitoring their presence in the environment is of crucial importance. The European roe deer’s interactions with its environment makes it a suitable bioindicator. Using UPLC-HRMS, this study aimed to quantify 15 PFAS in the muscle and liver of roe deer, in relation to the area of provenance, sex and age of the animals. Animals belonging to urbanized areas tended to have a higher PFAS concentration, although it was not statistically significant. The concentration for female and older individuals was higher than that in males and younger animals, respectively. In conclusion, this species might serve as a bioindicator due to its territorial behavior, although the reasons for why females showed higher concentrations of PFASs are not fully known and the higher concentrations in older animals are probably due to a decline in protective hepatic functions and longer exposure time.

**Abstract:**

Due to their physicochemical properties, per- and polyfluorinated alkyl substances (PFASs) persist and bioaccumulate in living organisms, causing adverse health effects. Since exposure to xenobiotics is influenced by factors related to both the living organism and the considered compounds, biomonitoring PFASs’ presence in the environment is of crucial importance. This study aimed to detect and quantify 15 PFASs in the muscle and liver of 40 roe deer from a specific area in Northern Italy by UPLC-HRMS. In the roe deer, liver PFAS concentrations were higher than those seen in muscle (*p* < 0.05). Although PFAS content in animals from urbanized areas was higher than those found in deer from rural areas, this difference was not statistically significant. In female roe deer, the concentration was higher than in males (*p* < 0.05); moreover, older animals showed higher concentrations of PFASs in the liver than younger animals (*p* < 0.05). In conclusion, the amount of PFASs was higher in tissues from roe deer belonging to urbanized areas, showing that this species might serve as a good bioindicator due to its territorial behavior. PFAS content was significantly higher in female roe deer, although the reason is not fully known. Finally, PFAS concentration was higher in the liver of older animals, probably due to compromised hepatic function.

## 1. Introduction

Per- and polyfluorinated alkyl substances (PFASs) are contaminants with amphiphilic characteristics and high thermal, chemical and biological stability, used in several industrial production processes since the 1940s [1,2]. Approximately 4700 compounds have been developed and are used daily as surface treatment chemicals, polymerization aids, non-stick cookware, pesticides and aqueous firefighting foams, and thus, they are frequently and commonly detectable in samples of wildlife and humans due to their high volatility [3,4]. As reported in several studies, these compounds, are ubiquitous in environmental matrices such as water, soil and sewage sludge [5]. Their concentration in surface, ground, marine and drinking water ranges from 0.71 ng/L (ppt) to 67 ng/L (ppt), and in soil, the number of PFASs analyzed ranged from 2 to 32, with a mean of 14. Total PFAS concentrations in soil ranged from <0.001 to 237 μg/kg [6,7].

Generally, due to their physicochemical properties, fluorochemical products exhibit high thermal, chemical and biological inertness and can resist degradation by acids, bases, oxidants, reductants, photolytic processes, microbes and metabolic processes [8]. Their environmental persistence seems associated with the length and the strength of their carbon–fluorine bond; the half-lives of PFASs are still not completely defined, but the estimated half-lives of perfluorooctanoic acid (PFOA) and perfluorooctanesulfonic acid (PFOS) in humans are 3.8 and 5.4 years, respectively [9]. Due to their resistance to biotic and abiotic degradation, PFASs, when present in water or soil, pose a potential risk of exposure to both humans and wildlife [2]. The main route of exposure for both humans and animals seems to be food intake, which appears to be the major factor contributing to background content in sera, and exposure to contaminated water and soils results in elevated blood levels of PFASs in both humans and animals [10].

A growing number of studies indicate that exposure to PFASs leads to severe health effects, such as the disruption of endocrine functions and metabolism [11], impairment of liver and thyroid hormones [12], impact on renal physiology [13] and bones [14], together with their immunotoxicity with immunosuppressive effects [15,16].

Due to these numerous health adverse effects, monitoring the presence of PFASs in the environment is of crucial importance. Exposure to these xenobiotics is influenced by several factors related both to the living organism and the focal compounds themselves. Thus, monitoring only the presence of these xenobiotics in the environment may be not sufficient to determine the real exposure of biota. As described by Rendón-Lugo et al. and Zhou et al. [17,18], the use of biomonitoring could be more useful than monitoring alone. Among wild mammals, the European roe deer (*Capreolus capreolus*) was described as a suitable bioindicator, as it owns unique behavioral characteristics such as small home ranges (16–80 ha) and its high behavioral plasticity allow it to live in a wide range of habitats, including those extensively used for human activities [19]. Roe deer feed mainly on leaves, young shoots, berries and grass. They typically feed on forages that are easily digestible and some studies have demonstrated that the content of pollutants in their muscles depends on their dietary intake [19,20,21,22]. Another noteworthy characteristic of the roe deer is its frequent cohabitation with livestock animals in pastures [23]. Detecting environmental pollutants in its tissues could potentially facilitate an evaluation of the pasture’s quality. In instances of significant contamination, this assessment could influence a choice to refrain from its utilization.

In light of the widespread occurrence of PFASs in the natural environment, and, by our estimation, the excellent suitability of roe deer as bioindicators for assessing the presence of these pollutants, the objective of this research is to detect and quantify PFAS concentrations in the muscle and liver tissues of roe deer hunted within a specific area in Northern Italy.

## 2. Materials and Methods

### 2.1. Chemicals and Reagents

The solvents used for this experiment were HPLC LC-MS-grade, methanol, acetonitrile and 25% ammonia solution purchased from VWR International S.r.l. (Radnor, PA, USA). The internal standards (ISs) MPFNA (perfluoro-[1,2,3,4,5 13C5] nonanoic acid) and MPFOS (perfluoro-[1,2,3,4-13C4] octanesulfonic acid), the native stock solution containing standards (IsoMix, 30 compounds) and ammonium formate were purchased from Wellington Laboratories Inc. (345 Southgate Drive, Guelph, ON, Canada). Water was purified by a Milli-Q system (Millipore, Merck KGaA, Darmstadt, Germany). Before the beginning of the experiment, individual stock standard solutions of ISs were prepared at the concentration of 1 mg/L in methanol and stored at −20 °C. Working solutions were prepared daily by dilution of the stock standard solutions in methanol.

### 2.2. Sample Collection and Preparation for the Extraction

**Figure 1:**
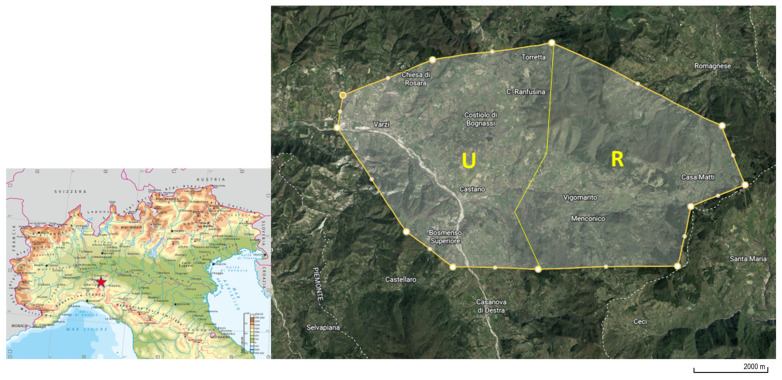
Map of the sampling area. U: urbanized; R: rural modified from [24].

This study drew on samples from 40 roe deer killed routinely by authorized hunters in a specific area of Northern Italy (Lombardy region, specifically Pavia province, Oltrepò Pavese, Figure 1). This study complied with Italian and international laws on animal experimentation and ethics (Animal Welfare Organisation of the University of Milan; Authorization No. 26_2022). The hunters signed the informed consent form included in the documentation to be submitted to the ethics committee. The hunting area of 5500 hectares was characterized by the presence of a rural area and an urbanized area. The rural part is mainly characterized by the presence of pastures and woods, while the urbanized area presents more anthropic activities and cultivated lands. Forty roe deer were enrolled in the study. The animals involved were grouped according to their age (20 young animals <2 years old of whom 10 were males and 10 females; 20 adult animals >2 years old of whom 10 were males and 10 females), sex (20 males, of whom 10 were young and 10 adult, and 20 females, of whom 10 were young and 10 adult) and area of origin (20 from the rural area, of whom 5 were young males, 5 young females, 5 adult males and 5 adult females, and 20 from the urbanized area of whom 5 were young males, 5 young females, 5 adult males and 5 adult females). Sampling procedures were performed during the slaughtering process at hunting meat processing plants; morphobiometric measures were registered and age was estimated through the evaluation of dental eruption and erosion. Figure S1 reports the distribution of animals in each estimated age class. From each animal, 100 g of muscle (*Longissimus lumborum et thoracis*, on the left side of the carcass) and 100 g of liver were collected. The samples were placed into glass conical centrifuge tubes of a volume of 50 mL (Corning Incorporated, Corning, NY, USA), immediately refrigerated at 4 °C, transported to the laboratory in a maximum amount of time of 12 h and then frozen at −20 °C until further analyses. Five grams of muscle or liver were transferred into a new glass 10 mL tube of acetonitrile and added for protein precipitation and PFAS extraction. Then, samples were homogenized by a T25 Digital ULTRA TURRAX^®^ (VWR International S.r.l., Radnor, PA, USA) for 1 min. To avoid cross-contamination, the blade of the homogenizer was washed with water, dried with paper, washed with 75% ethanol and rinsed with water after each sample. After the homogenization, the ISs were added to each sample in order to have a concentration of 5 ng/g of matrix. Samples were vortexed for 30 s and subsequently sonicated for 15 min. Immediately after, samples were centrifuged at 2500× *g* for 10 min at 4 °C. The obtained supernatant was transferred into a 15 mL glass tube and dried in a rotary vacuum centrifuge at 55 °C.

### 2.3. Extraction Procedure and UPLC-HRMS

The dried extract was suspended in 10 mL of purified Milli-Q water and underwent SPE extraction using the Strata PFAS (WAX/GCB), 200 mg/50 mg/6 mL, purchased by Phenomenex SRL (Torrance, CA, USA), for further purification and extraction under vacuum according to the conditions of a previously validated method [25]. At complete drying, the samples were resuspended in 100 µL of MeOH with a 100 µL mobile phase composed of 90% water with ammonium formate 20 mM and 10% of MeOH, centrifuged for 2 min at 23,500× *g* and transferred into vials for UPLC-HRMS. The instrumental method was performed according to the work recently published by Draghi et al. (2023) [26], which used a Vanquish Binary Pump, equipped with an autosampler and thermostat compartment for two columns (Thermo Fisher Scientific, Waltham, MA, USA), coupled with a Thermo Q Exactive OrbitrapTM (Thermo Fisher Scientific, Waltham, MA, USA) with a heated electrospray ionization source. The instrumental parameters of UPLC-HRMS are reported in the Supplementary Materials (Table S2). The post-run chromatograms and spectra were elaborated upon using the software XcaliburTM 4.3 (Thermo Fisher Scientific, Waltham, MA, USA) for data interpretation.

### 2.4. Method Validation

Validation was performed by following the SANTE updated regulation guidelines 11312/2021 [27], by evaluating the following parameters: selectivity, limit of detection (LOD), limit of quantification (LOQ) and matrix effect. by evaluating the interferences’ peaks in the blank samples close to the expected PFAS retention times, the selectivity of the method was confirmed. Eight procedural blanks were prepared without the matrix in each extraction session in order to avoid the misinterpretation of analytical results caused by the possible presence of traces of PFASs in the material used for sample extraction and purification. Through the analysis of 5 blank matrix samples, quality control assurance (QA/QC) was performed in order to determine the contribution of PFASs in the unfortified matrices and subtract the concentrations in the final calculations, if needed. By spiking the blank samples with the appropriate amount of standard mixture, matrix-matched calibration curves (10–100 pg g^−1^) were constructed. The following equations were used for calculate the LOD and LOQ limits: LOD = 3.3 SD/b and LOQ = 10 SD/b, where SD is the standard deviation of the intercept for low concentration levels and b is the slope of the regression line obtained from the principal calibration curve. The matrix effect is expressed as a percentage and was calculated by comparing the peak areas of PFASs that spiked after the extraction of a blank sample to the peak areas of standards in a solution mixture. The validation parameters are reported in Table S1.

### 2.5. Statistical Analysis

The statistical analysis was performed with the computer software Jamovi^®^ (Version 2.3). At first, the Shapiro–Wilk test was performed and it turned out that the data were not normally distributed. The age, gender, area and matrix were each categorized into two levels (<2 y.o./>2 y.o.; male/female; urbanized/rural; liver/muscle). In order to avoid a loss of statistical power caused by a reduction in sample size, samples that presented a complete absence of signal during the quantification were considered 0; samples below the LOQ were assigned an arbitrary value of ½ LOQ; and samples that presented a peak below the LOD were assigned an arbitrary value of ½ LOD. The percentage of samples >0 to the total of samples was used to calculate the detection frequency. The Mann–Whitney test was used to compare groups. The differences between groups were considered statistically significant with a *p*-value < 0.05, and tendencies were considered with a *p*-value ≤ 0.1.

## 3. Results

### 3.1. Distribution of Concentrations of the 15 Measured PFASs in Roe Deer Tissues and Concentrations in Liver and Muscle

In Table 1, the distribution of the concentrations of PFASs detected in tissues (muscle and liver) from the roe deer are reported as the percentage of samples above the LOQ.

The most frequently identified compounds were PFOA, PFNA and PFOS with percentages of 55.00%, 72.50% and 73.75%, respectively. FOSA was detected only in muscle samples. PFHxA, FOUEA, NADONA and N-MetFOSAA were never detected in this study; the other chemicals were identified in a range from 10 to 50% of samples.

**Figure 2:**
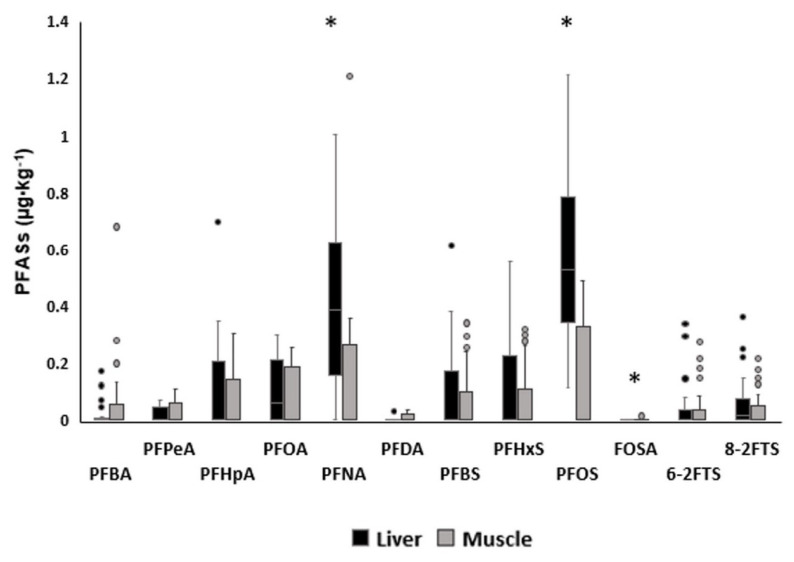
Comparison of PFAS content between the liver and muscle of roe deer; concentrations are expressed in µg·kg^−1^. *: statistically significant difference.

The data obtained from the analyses of the muscle and liver of roe deer from Oltrepò Pavese (Lombardy region, specifically Pavia province) are reported as the mean, median, minimum, maximum and percentile in Table S2 and Figure 2. To facilitate comparisons with the literature, the mean concentrations ± SD were used in Section 4.

Only three compounds showed statistically significant differences between the two matrices: PFNA was significantly higher in the liver than in the muscle, with median concentrations of 0.373 μg·kg^−1^ (Q25–Q75: 0.146–0.550) and 0.002 μg·kg^−1^ (Q25–Q75: 0–0.051) (*p* < 0.05). The median PFOS concentration identified in muscle was 0 μg·kg^−1^ (Q25–Q75: 0–0.277) and in the liver 0.526 μg·kg^−1^ (Q25–Q75: 0.351–0.782), therefore being significantly higher in the liver.

### 3.2. Comparison between Urbanized and Rural Areas in Liver and Muscle Concentrations of PFASs from Roe Deer

**Figure 3:**
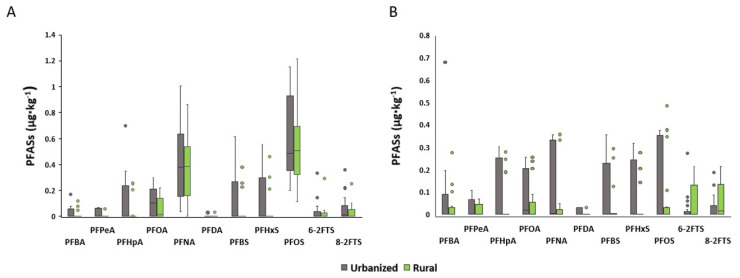
Comparison between urbanized and rural areas of PFAS content in the (**A**) liver and (**B**) muscle of roe deer; concentrations are expressed in µg·kg^−1^.

The liver and muscle concentrations of the detected PFASs in the urbanized and rural areas are shown in Figure 3. No statistically significant differences between areas were identified. Tables S3 and S4 present the mean concentrations with the standard deviations, medians and percentiles, minimum and maximum levels, and detection frequencies of the PFASs in the liver and muscle categorized according to the roe deer’s area of origin.

### 3.3. Comparison between Sexes in Liver and Muscle Concentrations of PFASs

**Figure 4:**
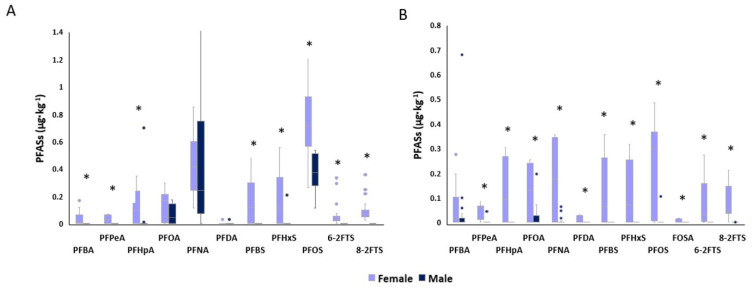
Comparison between male and female roe deer PFAS content in the (**A**) liver and (**B**) muscle; concentrations are expressed in µg·kg^−1^. *: statistically significant difference.

The mean concentrations of the PFASs detected in the tissues of male and female roe deer are shown in Tables S5 and S6, and the comparison between the two sexes is shown in Figure 4. In the liver (Figure 4A), PFBA, PFPeA, PFBS, 6-2FTS and 8-2FTS were not detected in males. PFHpA’s median concentration in females was 0.1 µg·kg^−1^ (Q25–Q75: 0–0.237) and 0 µg·kg^−1^ (Q25–Q75: 0–0) in males, being significantly higher in females. The content of PFHxS in the liver was higher in females (*p* < 0.05) than in males with median concentrations of 0.113 µg·kg^−1^ (Q25–Q75: 0–0.325) and 0 µg·kg^−1^ (Q25–Q75: 0–0), respectively. PFOS median concentrations were significantly higher in females, (0.713 µg·kg^−1^ with Q25–Q75: 0.576–0.877) than in males (0.371 µg·kg^−1^ with Q25–Q75: 0.297–0.492).

In the muscle, PFHxA was not detected in either sex. As can be seen in Figure 4B, PFHpA, PFDA, PFBS, PFHxS, FOSA, 6-2FTS and 8-2FTS were not identified in males. PFBA concentration in the muscle of females was significantly higher (0.017 µg·kg^−1^ with Q25–Q75: 0–0.097) than in males (0 µg·kg^−1^ with Q25–Q75: 0–0.005). Even for PFPeA, the concentration was higher in females with a median quantification of 00.059 µg·kg^−1^ (Q25–Q75: 0.033–0.066) compared to 0 µg·kg^−1^ (Q25–Q75: 0–0) registered in males. The median concentration of PFOA in muscle was 0.131 µg·kg^−1^ (Q25–Q75: 0–0.238) and 0 µg·kg^−1^ (Q25–Q75: 0–0.009) in females and males, respectively, being higher in females. For PFNA, the median concentration registered in the muscle of female roe deer was 0.175 µg·kg^−1^ (Q25–Q75: 0.004–0.342), whereas in males 0 µg·kg^−1^ (Q25–Q75: 0–0.002), and thus significantly lower. PFOS concentration was significantly higher in females (0.301 µg·kg^−1^ with Q25–Q75: 0.011–0.366) than in males (0 µg·kg^−1^ with Q25–Q75: 0–0).

### 3.4. Comparison between Age Classes in Liver and Muscle Concentrations of PFASs in Roe Deer Aged < 2 y.o. and Aged > 2 y.o.

**Figure 5:**
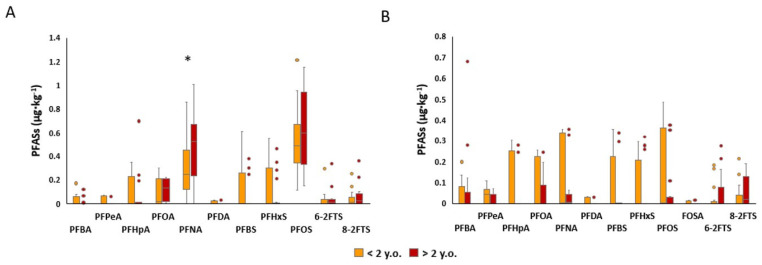
Comparison between PFAS content in the (**A**) liver and (**B**) muscle of roe deer aged < 2 y.o. and > 2 y.o.; concentrations are expressed in µg·kg^−1^. *: statistically significant difference.

The median concentrations, percentiles, minimum and maximum levels and means ± SD of the PFASs detected in the tissues of the roe deer grouped according to the age of the animals are presented in Tables S7 and S8. The comparison between age classes is shown in Figure 5. In the liver, PFNA concentration was significantly higher in animals aged > 2 y.o., with a median concentration of 0.523 µg·kg^−1^ (Q25–Q75: 0.2516–0.653) compared to 0.243 µg·kg^−1^ (Q25–Q75: 0.126–0.414) (<2 y.o.).

In the muscle (Figure 5B), no statistically significant differences were determined between the two age groups.

## 4. Discussion

### 4.1. PFAS Concentrations in Roe Deer Tissues

This study marked a first instance of quantifying the presence of PFASs within the focal area. As such, we initially attempted to contextualize our results by comparing them with those obtained in studies conducted in other areas or on other species.

As reported in several papers, PFASs are primarily distributed in the serum, liver and kidneys [28,29], instead of similarly to hydrophobic contaminants such as PAHs, PCBs, etc., which are mainly accumulated in adipose tissues [30]. Tissue-to-serum partition coefficients of PFASs vary by chemical type; as reported by other authors, while PFOS, PFOA and PFBS preferentially bioaccumulate in protein-rich compartments such as the liver and blood, PFBA and PFHxS are mainly distributed in the serum [31,32].

As expected and reported in other studies, PFNA and PFOS were significantly higher in the liver than in the muscle. Other compounds did not show any statistical difference but a trend of higher concentration in the liver than in the muscle is reported (Figure 2). These findings are in accordance with recent studies on beef cattle, in which the concentration of PFASs in the liver was higher than in muscle [33], and in pigs, in which PFASs were found in the liver but were not measurable in other tissues [34]. It is known that PFASs exhibit a high affinity for plasma albumin and thus an explanation for the major accumulation of PFASs in the liver is that the liver is the synthesis site for this protein [35]. A recent report by the EFSA states that PFOS and PFOA concentration is particularly high in liver samples from game mammals. For example, in the wild boar liver, the mean concentration of PFOS was 215 µg·kg**^−^**^1^ and 8.18 µg·kg**^−^**^1^ for PFOA [36], while in our study, their mean concentrations were much lower: 0.66 µg·kg**^−^**^1^ for PFOS and 0.15 µg·kg**^−^**^1^ for PFOA. These differences could be explained by the differences between species, particularly concerning their feeding habits, digestive physiology features and home ranges. The concentrations of PFASs found in the tissues of the roe deer sampled for our study were lower than those found in a study by Falk et al. (2012) [1]. These differences may be attributed to the different sampling areas used. Indeed, their study was conducted in Germany, in a larger area than ours, which covered different habitats, including forested, agricultural and suburban areas. In addition, the sampling was carried out in a completely different historical period (1989–2010) when laws aiming to reduce PFAS use had not yet been enacted. Moreover, in our case, the spatiotemporal window was smaller (only during a hunting season, actually one calendar year) [1].

To our knowledge, there have not been any studies conducted on PFAS concentration in roe deer muscle, but the content detected in this study was lower compared to other species, such as the duck [37], cattle [33] and pig [38].

### 4.2. Comparison of PFAS Content in Roe Deer Tissues Belonging to Urbanized and Rural Areas

Aiming to use the roe deer as a bioindicator of the presence of PFASs in a specific area of Northern Italy, their tissue concentration was compared between the group of animals from the urbanized area and the rural area. As reported in Section 2, the two areas differ in anthropogenic activities, including agricultural activities. As described in numerous studies, PFASs are contained in substances such as herbicides and pesticides widely used in these activities [25], so a difference in concentration in the tissues of roe deer belonging to rural and urbanized areas was expected. The mean concentrations of PFASs detected in the urbanized area deer were not significantly higher than in the rural area deer, even if, as shown in Figure 3, all compounds tended to be higher (*p* ≤ 0.1) in animals from the urbanized area. The lack of statistically significant differences could be due to the large standard deviations and the presence of some outliers in the rural areas. As reported by Death et al. animals belonging from peri-urban areas may have higher PFAS tissue concentrations due to a higher density of the source of exposure, such as industries and landfills [39]. Spatial differences in PFAS content based on sampling location were observed. In a study conducted by Falk et al. on roe deer in Germany, the content of PFASs in liver samples was higher in animals belonging to ecosystems close to conurbations compared to those from agrarian ecosystems, proving that the regional differences in PFAS concentrations is related to differences in environmental exposure [1]. Furthermore, our results overlap with other work on roe deer and red deer in which the concentrations of various environmental contaminants were compared between different areas, being higher in animals from the area with the most anthropogenic activities [24,40]. Although the average concentrations found in this study tended to be higher in the urbanized area, as shown in Tables S3 and S4, for some of the compounds, the detection frequencies and maximum levels were higher in the rural area. This result seems to be controversial, but, as reported by Sunderland et al., one of the main sources of PFASs is flame retardants and aqueous film-forming foams (AFFFs) used for firefighting purposes [41]. In this case, the rural area was subjected to fires several times, explaining the higher detection frequencies and maximum levels identified for PFOA, PFHpA and PFNA. Furthermore, it is worth remembering that one of the fundamental characteristics that make roe deer an excellent bioindicator is its territorial behavior and small home range [19]. Indeed, these species-specific characteristics are what enable the identification of hot spots of contamination and could explain the high number of outliers in the rural area.

### 4.3. Liver and Muscle Concentrations of PFASs in Males and Females and Comparison between Sexes

The amount of PFASs detected in the liver and muscle from female roe deer was significantly higher than that detected in roe bucks (Figure 4 and Tables S4 and S5). In the literature, it is reported that usually, females tend to accumulate higher amounts of xenobiotics in their tissues [42]. These findings are mainly related to PCBs, dioxins and several pesticides, and it has been assumed by several authors that those results are related to their differences in body composition. Indeed, females generally have a higher proportion of bodily adipose tissue than males which can act as a reservoir for lipophilic xenobiotics [43,44]. PFASs, in contrast with the hydrophobic contaminants mentioned above, are known to be mainly distributed in the serum, liver and kidneys; thus, their bioaccumulation potential cannot be predicted by traditional approaches and this aspect needs to be clarified by further studies.

In addition to differences in body composition, hormonal factors can also play a role in sex differences in xenobiotic accumulation. For example, estrogen and progesterone can influence the absorption, distribution and elimination of xenobiotics in the body [45]. Studies have shown that some xenobiotics can interact with these hormones, potentially leading to altered metabolism and accumulation in tissues. For example, estrogen has been shown to increase the expression of certain proteins that transport PFASs into cells. Indeed, PFASs bind peroxisome proliferator-activated receptors which are directly involved in the conversion of androgens to estrogens, potentially leading to higher levels of PFASs in female tissues [46,47]. On the other hand, testosterone may have a protective effect against some xenobiotics, potentially due to its antioxidative properties [48]. Another possible explanation is attributable to their reproductive physiology. For example, during pregnancy and lactation, females may mobilize fat stores that contain accumulated xenobiotics, potentially leading to increased levels in blood and tissues [49,50]. This may be particularly relevant for lipophilic compounds that tend to accumulate in adipose tissue but not for PFASs. However, in a study conducted by Mondal et al., lactation seems to have been a way for PFASs to be excreted, and this could explain the mobilization of these compounds during this reproductive phase, justifying our findings [51]. It is indeed necessary to consider that the female roe deer involved in this study were hunted during their last period of lactation and resumption of pregnancy after diapause (the hunting period for females is from January until March). It is also important to note that the exact reasons for the higher levels of PFASs in female tissues are still being studied and debated among researchers, and more research is needed to fully understand the sex differences in PFAS exposure and accumulation.

### 4.4. Liver and Muscle Concentrations of PFASs in Roe Deer Aged < 2 y.o. and Aged > 2 y.o. and Comparison between Age Classes

Only PFNA was significantly higher in the liver of older animals compared to younger animals. Meanwhile, in the case of PFOA, PFOS and telomers, no statistically significant differences were highlighted, but a tendency to be higher in older animals was present (Figure 5). Other analyzed compounds were detected in similar amounts between the two classes of age, as reported in Tables S7 and S8. The roe deer used in this study were grouped according to two estimated age classes (young < 2 y.o. and adult > 2 y.o.). Those belonging to the >2 y.o. class had an average age of 4 years and the others of 0.8 years, and in the adult age class, a higher span of ages was included. Considering that the maximum age that can be estimated by dental eruption and erosion is 6 years, roe deer in the age range of 4 to 6 years are considered elderly [52,53], and aging is a possible explanation for the higher accumulation of xenobiotics in the liver of animals in this age class. In several studies, it has been reported that aging accompanies changes in metabolism with a consequent change in the biotransformation of xenobiotics due to liver function decline [54,55]. Indeed, the liver produces xenobiotic metabolizing enzymes and if it is not functioning at full capacity, toxic substances can build up in the body, leading to liver toxicity [56]. For example, the liver may become less efficient at breaking down certain toxins, leading to the bioaccumulation of these substances in the organism. One of the causes that leads to liver function decline is the reduction in the liver’s ability to regenerate hepatocytes. It is well known that the liver has the ability to replace damaged cells with new ones, but if this activity is lacking, the difficulty of recovering the damage caused by xenobiotics increases. It is worth noting that liver toxicity can occur in animals of all ages, but the risk may be higher in older animals due to these factors [57,58]. 

Taking into account that PFASs are bioaccumulative substances with a high half-life, another factor that may have influenced the higher amount of xenobiotics accumulated in the liver is that older animals had been chronically exposed for a longer time [57]. Moreover, in this study, despite the lack of statistical significance, probably due to the presence of outliers, a tendentially higher concentration could be observed in the muscle of younger animals. There are several possible explanations for the higher levels of toxic and trace elements in the young animals’ muscles. From a physiological point of view, young animals have higher metabolic rates than older animals, which means that they may absorb and accumulate more elements from their environment [59]. Moreover, considering that half of the young animals involved in this study were less than one year old, it is plausible to think that they may also have had less efficient mechanisms for excreting these elements, which could have contributed to their higher levels of accumulation in their muscles [60].

## 5. Conclusions

In conclusion, it can be said that the content of PFASs was higher in the liver than in the muscle, which demonstrated the compounds’ higher affinity to proteins synthesized by this metabolizing organ. The amount of PFASs tended to be higher in the tissues obtained from roe deer from the urbanized area, showing that roe deer could be a promising bioindicator of PFAS presence due to its territorial behaviour and condensed home range. In line with other studies, the PFAS content was significantly higher in females, albeit the cause of this is not yet fully understood. Finally, PFAS content was higher in the liver of older animals, probably due to the higher bioaccumulation of PFASs in tissues of older animals subsequent to a longer exposure time and the decline of their hepatic functions due to aging.

## Figures and Tables

**Table 1:** Distribution of concentrations of 15 PFASs detected in tissues (muscle and liver) from roe deer sampled in the area of Oltrepò Pavese (Lombardy region, specifically Pavia province) expressed in μg/kg. LOD: limit of detection; LOQ: limit of quantification; N.D.: not detected.

Compound		No. of Samples	LOD	LOQ	% Samples > LOQ
**PFBA**	Perfluorobutanoic acid	80	0.0006	0.0019	32.5
**PFPeA**	Perfluoropentanoic acid	80	0.0004	0.0012	33.75
**PFHxA**	Perfluorohexanoic acid	80	0.0027	0.0082	N.D.
**PFHpA**	Perfluoroheptanoic acid	80	0.0002	0.0007	30.0
**PFOA**	Perfluorooctanoic acid	80	0.0005	0.0014	55.0
**PFNA**	Perfluorononanoic acid	80	0.0017	0.0051	72.5
**PFDA**	Perfluorodecanoic acid	80	0.0002	0.0006	22.5
**FOUEA**	2H-perfluoro-2-decenoic acid	80	0.0009	0.0027	N.D.
**NADONA**	Sodium dodecafluoro-3H-4,8--dioxanonanoate	80	0.0002	0.0005	N.D.
**PFBS**	Perfluorobutanesulfonic acid	80	0.0069	0.0210	26.25
**PFHxS**	Perfluorohexanesulfonic acid	80	0.0002	0.0007	27.5
**PFOS**	Perfluorooctanesulfonic acid	80	0.0005	0.0014	73.75
**FOSA**	Perfluorooctanesulfonamide	80	0.0021	0.0064	11.25
**6-2FTS**	6:2-fluorotelomersulfonic acid	80	0.0004	0.0012	43.75
**8-2FTS**	8:2-fluorotelomersulfonic acid	80	0.0004	0.0013	50

## Data Availability

The data presented in this study are available in the article and Supplementary Materials. Further information is available upon request from the corresponding author.

## Supplementary Materials

The following supporting information can be downloaded at: https://www.mdpi.com/article/10.3390/ani14040529/s1, Figure S1: Age classes and their distribution; Table S1: Validation parameters of UPLC-HRMS analysis and instrumental parameter information; Table S2: Concentrations of PFASs in liver and muscle tissue and comparison between matrices; Table S3: PFASs detected in the liver of roe deer, compared between urbanized and rural areas; Table S4: PFASs detected in the muscle of roe deer, compared between urbanized and rural areas; Table S5: PFASs detected in the liver of roe deer, compared between sexes; Table S6: PFASs detected in the muscle of roe deer, compared between sexes; Table S7: PFASs detected in the liver of roe deer, compared between age classes; Table S8: PFASs detected in the muscle of roe deer, compared between age classes.

## References

[1] Falk S., Brunn H., Schröter-Kermani C., Failing K., Georgii S., Tarricone K., Stahl T. (2012). Temporal and Spatial Trends of Perfluoroalkyl Substances in Liver of Roe Deer (*Capreolus capreolus*). Environ. Pollut..

[2] Jha G., Kankarla V., McLennon E., Pal S., Sihi D., Dari B., Diaz D., Nocco M. (2021). Per- and Polyfluoroalkyl Substances (PFAS) in Integrated Crop-Livestock Systems: Environmental Exposure and Human Health Risks. Int. J. Environ. Res. Public. Health.

[3] Rayne S., Forest K. (2009). Perfluoroalkyl Sulfonic and Carboxylic Acids: A Critical Review of Physicochemical Properties, Levels and Patterns in Waters and Wastewaters, and Treatment Methods. J. Environ. Sci. Health A Toxic Hazard. Subst. Environ. Eng..

[4] Hammer J., Endo S. (2022). Volatility and Nonspecific van Der Waals Interaction Properties of Per- and Polyfluoroalkyl Substances (PFAS): Evaluation Using Hexadecane/Air Partition Coefficients. Environ. Sci. Technol..

[5] Clarke B.O., Smith S.R. (2011). Review of “emerging” Organic Contaminants in Biosolids and Assessment of International Research Priorities for the Agricultural Use of Biosolids. Environ. Int..

[6] Al Amin M., Sobhani Z., Liu Y., Dharmaraja R., Chadalavada S., Naidu R., Chalker J.M., Fang C. (2020). Recent Advances in the Analysis of Per- and Polyfluoroalkyl Substances (PFAS): A Review. Environ. Technol. Innov..

[7] Brusseau M.L., Anderson R.H., Guo B. (2020). PFAS Concentrations in Soils: Background Levels versus Contaminated Sites. Sci. Total Environ..

[8] Parsons J.R., Sáez M., Dolfing J., de Voogt P. (2008). Biodegradation of Perfluorinated Compounds. Rev. Environ. Contam. Toxicol..

[9] Takacs M.L., Abbott B.D. (2007). Activation of Mouse and Human Peroxisome Proliferator-Activated Receptors (Alpha, Beta/Delta, Gamma) by Perfluorooctanoic Acid and Perfluorooctane Sulfonate. Toxicol. Sci..

[10] Vestergren R., Cousins I.T. (2009). Tracking the Pathways of Human Exposure to Perfluorocarboxylates. Environ. Sci. Technol..

[11] Christensen K.Y., Raymond M., Meiman J. (2019). Perfluoroalkyl Substances and Metabolic Syndrome. Int. J. Hyg. Environ. Health.

[12] Coperchini F., Croce L., Ricci G., Magri F., Rotondi M., Imbriani M., Chiovato L. (2021). Thyroid Disrupting Effects of Old and New Generation PFAS. Front. Endocrinol..

[13] Stanifer J.W., Stapleton H.M., Souma T., Wittmer A., Zhao X., Boulware L.E. (2018). Perfluorinated Chemicals as Emerging Environmental Threats to Kidney Health: A Scoping Review. Clin. J. Am. Soc. Nephrol..

[14] Jeddy Z., Tobias J.H., Taylor E.V., Northstone K., Flanders W.D., Hartman T.J. (2018). Prenatal Concentrations of Perfluoroalkyl Substances and Bone Health in British Girls at Age 17. Arch. Osteoporos..

[15] Ehrlich V., Bil W., Vandebriel R., Granum B., Luijten M., Lindeman B., Grandjean P., Kaiser A.M., Hauzenberger I., Hartmann C. (2023). Consideration of Pathways for Immunotoxicity of Per- and Polyfluoroalkyl Substances (PFAS). Environ. Health.

[16] Liang L., Pan Y., Bin L., Liu Y., Huang W., Li R., Lai K.P. (2022). Immunotoxicity Mechanisms of Perfluorinated Compounds PFOA and PFOS. Chemosphere.

[17] Rendón-Lugo A.N., Santiago P., Puente-Lee I., León-Paniagua L. (2017). Permeability of Hair to Cadmium, Copper and Lead in Five Species of Terrestrial Mammals and Implications in Biomonitoring. Environ. Monit. Assess..

[18] Zhou Q., Zhang J., Fu J., Shi J., Jiang G. (2008). Biomonitoring: An Appealing Tool for Assessment of Metal Pollution in the Aquatic Ecosystem. Anal. Chim. Acta.

[19] Cygan-Szczegielniak D., Stasiak K. (2022). Effects of Age and Sex on the Content of Heavy Metals in the Hair, Liver and the Longissimus Lumborum Muscle of Roe Deer *Capreolus capreolus* L.. Environ. Sci. Pollut. Res. Int..

[20] Tixier H., Duncan P., Scehovic J., Yani A., Gleizes M., Lila M. (1997). Food Selection by European Roe Deer (*Capreolus capreolus*): Effects of Plant Chemistry, and Consequences for the Nutritional Value of Their Diets. J. Zool..

[21] Lehel J., Laczay P., Gyurcsó A., Jánoska F., Majoros S., Lányi K., Marosán M. (2016). Toxic Heavy Metals in the Muscle of Roe Deer (*Capreolus Capreolus*)--Food Toxicological Significance. Environ. Sci. Pollut. Res. Int..

[22] Pavlovic R., Draghi S., Pellegrini A., Silva C.F., Di Cesare F., Curone G., Arioli F., Fidani M. (2024). High-Resolution Mass Spectrometry Non-Targeted Detection of Per- and Polyfluoroalkyl Substances in Roe Deer (*Capreolus capreolus*). Molecules.

[23] Varela-Castro L., Sevilla I.A., Payne A., Gilot-Fromont E., Barral M. (2021). Interaction Patterns between Wildlife and Cattle Reveal Opportunities for Mycobacteria Transmission in Farms from North-Eastern Atlantic Iberian Peninsula. Animals.

[24] Draghi S., Agradi S., Riva F., Tarhan D., Bilgiç B., Dokuzeylül B., Ercan A.M., Or M.E., Brecchia G., Vigo D. (2023). Roe Deer (*Capreolus capreolus*) Hair as a Bioindicator for the Environmental Presence of Toxic and Trace Elements. Toxics.

[25] Chiesa L.M., Pavlovic R., Arioli F., Nobile M., Di Cesare F., Mosconi G., Falletta E., Malandra R., Panseri S. (2022). Presence of Perfluoroalkyl Substances in Mediterranean Sea and North Italian Lake Fish Addressed to Italian Consumer. Int. J. Food Sci. Technol..

[26] Draghi S., Pavlovic R., Pellegrini A., Fidani M., Riva F., Brecchia G., Agradi S., Arioli F., Vigo D., Di Cesare F. (2023). First Investigation of the Physiological Distribution of Legacy and Emerging Perfluoroalkyl Substances in Raw Bovine Milk According to the Component Fraction. Foods.

[27] Pihlström T., Fernández-Alba A.R., Ferrer Amate C., Erecius Poulsen M., Lippold R., Carrasco Cabrera L., Pelosi P., Valverde A., Unterluggauer H., Mol H. (2017). Analytical Quality Control and Method Validation Procedures for Pesticide Residues Analysis in Food and Feed. Sante.

[28] Blake B.E., Fenton S.E. (2020). Early Life Exposure to Per- and Polyfluoroalkyl Substances (PFAS) and Latent Health Outcomes: A Review Including the Placenta as a Target Tissue and Possible Driver of Peri- and Postnatal Effects. Toxicology.

[29] Pérez F., Nadal M., Navarro-Ortega A., Fàbrega F., Domingo J.L., Barceló D., Farré M. (2013). Accumulation of Perfluoroalkyl Substances in Human Tissues. Environ. Int..

[30] La Merrill M., Emond C., Kim M.J., Antignac J.P., Le Bizec B., Clément K., Birnbaum L.S., Barouki R. (2013). Toxicological Function of Adipose Tissue: Focus on Persistent Organic Pollutants. Environ. Health Perspect..

[31] Kelly B.C., Ikonomou M.G., Blair J.D., Surridge B., Hoover D., Grace R., Gobas F.A.P.C. (2009). Perfluoroalkyl Contaminants in an Arctic Marine Food Web: Trophic Magnification and Wildlife Exposure. Environ. Sci. Technol..

[32] Pizzurro D.M., Seeley M., Kerper L.E., Beck B.D. (2019). Interspecies Differences in Perfluoroalkyl Substances (PFAS) Toxicokinetics and Application to Health-Based Criteria. Regul. Toxicol. Pharmacol..

[33] Wang G., Lu J., Xing Z., Li S., Liu Z., Tong Y. (2017). Occurrence, Distribution, and Risk Assessment of Perfluoroalkyl Acids (PFAAs) in Muscle and Liver of Cattle in Xinjiang, China. Int. J. Environ. Res. Public. Health.

[34] Numata J., Kowalczyk J., Adolphs J., Ehlers S., Schafft H., Fuerst P., Müller-Graf C., Lahrssen-Wiederholt M., Greiner M. (2014). Toxicokinetics of Seven Perfluoroalkyl Sulfonic and Carboxylic Acids in Pigs Fed a Contaminated Diet. J. Agric. Food Chem..

[35] Lau C. (2015). Perfluorinated Compounds: An Overview. Molecular and Integrative Toxicology.

[36] Knutsen H.K., Alexander J., Barregård L., Bignami M., Brüschweiler B., Ceccatelli S., Cottrill B., Dinovi M., Edler L., Grasl-Kraupp B. (2018). Risk to Human Health Related to the Presence of Perfluorooctane Sulfonic Acid and Perfluorooctanoic Acid in Food. EFSA J..

[37] Sharp S., Sardiña P., Metzeling L., McKenzie R., Leahy P., Menkhorst P., Hinwood A. (2021). Per- and Polyfluoroalkyl Substances in Ducks and the Relationship with Concentrations in Water, Sediment, and Soil. Environ. Toxicol. Chem..

[38] Chen W.L., Bai F.Y., Chang Y.C., Chen P.C., Chen C.Y. (2018). Concentrations of Perfluoroalkyl Substances in Foods and the Dietary Exposure among Taiwan General Population and Pregnant Women. J. Food Drug Anal..

[39] Death C., Bell C., Champness D., Milne C., Reichman S., Hagen T. (2021). Per- and Polyfluoroalkyl Substances (PFAS) in Livestock and Game Species: A Review. Sci. Total Environ..

[40] Cygan-Szczegielniak D., Stanek M., Stasiak K., Roślewska A., Janicki B. (2018). The Content of Mineral Elements and Heavy Metals in the Hair of Red Deer (*Cervus elaphus* L.) from Selected Regions of Poland. Folia Biol..

[41] Sunderland E.M., Hu X.C., Dassuncao C., Tokranov A.K., Wagner C.C., Allen J.G. (2019). A Review of the Pathways of Human Exposure to Poly- and Perfluoroalkyl Substances (PFASs) and Present Understanding of Health Effects. J. Expo. Sci. Environ. Epidemiol..

[42] Aluc Y., Ekici H. (2019). Investigation of Heavy Metal Levels in Blood Samples of Three Cattle Breeds in Turkey. Bull. Environ. Contam. Toxicol..

[43] Karastergiou K., Smith S.R., Greenberg A.S., Fried S.K. (2012). Sex Differences in Human Adipose Tissues—The Biology of Pear Shape. Biol. Sex. Differ..

[44] Jandacek R.J., Tso P. (2001). Factors Affecting the Storage and Excretion of Toxic Lipophilic Xenobiotics. Lipids.

[45] Kohalmy K., Vrzal R. (2011). Regulation of Phase II Biotransformation Enzymes by Steroid Hormones. Curr. Drug Metab..

[46] Ding N., Harlow S.D., Randolph J.F., Loch-Caruso R., Park S.K. (2020). Perfluoroalkyl and Polyfluoroalkyl Substances (PFAS) and Their Effects on the Ovary. Hum. Reprod. Update.

[47] Fan W.Q., Yanase T., Morinaga H., Mu Y.M., Nomura M., Okabe T., Goto K., Harada N., Nawata H. (2005). Activation of Peroxisome Proliferator-Activated Receptor-Gamma and Retinoid X Receptor Inhibits Aromatase Transcription via Nuclear Factor-KappaB. Endocrinology.

[48] Cruz-Topete D., Dominic P., Stokes K.Y. (2020). Uncovering Sex-Specific Mechanisms of Action of Testosterone and Redox Balance. Redox Biol..

[49] Glynn A., Larsdotter M., Aune M., Darnerud P.O., Bjerselius R., Bergman Å. (2011). Changes in Serum Concentrations of Polychlorinated Biphenyls (PCBs), Hydroxylated PCB Metabolites and Pentachlorophenol during Pregnancy. Chemosphere.

[50] Stawarz R., Formicki G., Massányi P. (2007). Daily Fluctuations and Distribution of Xenobiotics, Nutritional and Biogenic Elements in Human Milk in Southern Poland. J. Environ. Sci. Health A Toxic Hazard. Subst. Environ. Eng..

[51] Mondal D., Weldon R.H., Armstrong B.G., Gibson L.J., Lopez-Espinosa M.J., Shin H.M., Fletcher T. (2014). Breastfeeding: A Potential Excretion Route for Mothers and Implications for Infant Exposure to Perfluoroalkyl Acids. Environ. Health Perspect..

[52] Hewison A.J.M., Vincent J.P., Angibault J.M., Delorme D., Van Laere G., Gaillard J.M. (1999). Tests of Estimation of Age from Tooth Wear on Roe Deer of Known Age: Variation within and among Populations. Can. J. Zool..

[53] Tomé C., Vigne J., Tome C., Vigne J. (2003). Roe Deer (*Capreolus Capreolus*) Age at Death Estimates: New Methods and Modern Reference Data for Tooth Eruption and Wear, and for Epiphyseal Fusion. Archaeofauna Int. J. Archaeozoology.

[54] Shi S., Klotz U. (2011). Age-Related Changes in Pharmacokinetics. Curr. Drug Metab..

[55] Vyskočilová E., Szotáková B., Skálová L., Bártíková H., Hlaváčová J., Boušová I. (2013). Age-Related Changes in Hepatic Activity and Expression of Detoxification Enzymes in Male Rats. Biomed. Res. Int..

[56] Corton J.C., Lee J.S., Liu J., Ren H., Vallanat B., DeVito M. (2022). Determinants of Gene Expression in the Human Liver: Impact of Aging and Sex on Xenobiotic Metabolism. Exp. Gerontol..

[57] Hunt N.J., Kang S.W. (Sophie), Lockwood G.P., Le Couteur D.G., Cogger V.C. (2019). Hallmarks of Aging in the Liver. Comput. Struct. Biotechnol. J..

[58] Yun K.U., Oh S.J., Oh J.M., Kang K.W., Myung C.S., Song G.Y., Kim B.H., Kim S.K. (2010). Age-Related Changes in Hepatic Expression and Activity of Cytochrome P450 in Male Rats. Arch. Toxicol..

[59] Riyazuddin R., Nisha N., Ejaz B., Khan M.I.R., Kumar M., Ramteke P.W., Gupta R. (2021). A Comprehensive Review on the Heavy Metal Toxicity and Sequestration in Plants. Biomolecules.

[60] Lee E.Y., Liszewski M.C., Gee M.S., Daltro P., Restrepo R. (2020). Pediatric Body MRI: A Comprehensive, Multidisciplinary Guide.

