# Supplementary material for: Influence of Area, Age and Sex on Per- and Polyfluorinated Alkyl Substances Detected in Roe Deer Muscle and Liver from Selected Areas of Northern Italy
Source: Animals (Basel). 2024 Feb 6;14(4):529. doi: 10.3390/ani14040529 (PMC10885967; doi:10.3390/ani14040529)
Supplement: Supplementary file 1 [file animals-14-00529-s001.zip › Supplementary materials.pdf]

Figure S1: Age classes and their distribution. Estimated age is expressed in in months for animals < 2 y.o. and in years for animals > 2 y.o.

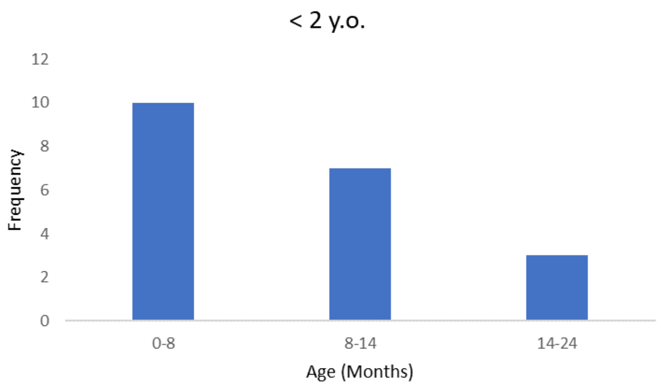

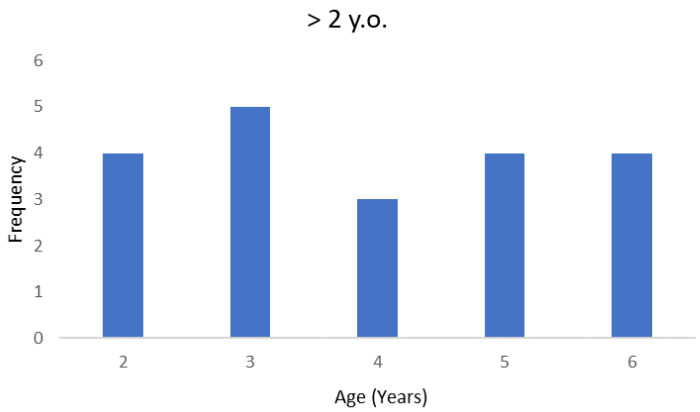

Table S1: Validation parameters of UPLC-HRMS analysis and instrumental parameters information.

Compound	Formula	Parent Exact Mass m/z	RT (min)	LOD (pg/g)	LOQ (pg/g)	Linearity	R^2	Recovery (%)	Matrix Effect (%)	CV Intraday (%)	CV Interday (%)
PFBA	C4HF7O2	213	3.71	0.615116	1.863989	y = 0.0706x + 0.0067	0.978	108	83	11	19
PFPeA	C5HF9O	263	9.64	0.402452	1.219553	y = 1.1245x - 0.0501	0.979	101	82	12	15
PFHxA	C6HF11O2	313	13.37	2.715292	8.228159	y = 0.1497x + 0.0012	0.999	98	100	10	18
PFHpA	C7HF13O2	363	15.53	0.247166	0.748987	y = 0.1842x + 0.0014	0.999	89	109	10	16
PFOA	C8HF15O2	413	17.05	0.456216	1.382472	y = 0.2022x - 0.0061	0.999	97	85	11	17
PFNA	C9HF17O2	463	18.27	1.680316	5.091868	y = 0.1088x + 0.0004	0.999	101	102	12	20
PFDA	C10HF19O2	513	21.92	0.208077	0.630537	y = 0.1674x - 0.005	0.972	106	99	11	15
FOUEA	C10H2F16O	457	18.73	0.8751	2.651818	y = 0.0384x - 0.0001	0.998	100	105	13	17
NaDONA	C7HF12NaO4	377	15.75	0.166717	0.505202	y = 0.2671x + 0.003	0.997	102	116	14	20
PFBS	C4HF9O3S	299	10.90	6.925848	20.98742	y = 0.3081x + 0.0037	0.999	98	90	9	16
PFHxS	C6HF13O3S	399	15.67	0.242975	0.736289	y = 0.2344 + 0.0072	0.976	103	114	12	20
PFOS	C8HF17O3S	499	18.24	0.47006	1.424425	y = 0.1144x + 0.0021	0.999	97	101	11	18
FOSA	C8H2F17NO2S	498	20.60	2.102951	6.37258	y = 0.0494x - 0.0006	0.985	100	89	10	18
6-2FTS	C8H5F13O3S	427	16.96	0.401824	1.217649	y = 0.0628x + 0.0018	0.980	99	95	10	15
8-2FTS	C10H4F17O3S	527	21.15	0.433713	1.314282	y = 0.1068x - 5E-05	0.999	97	100	12	17
N-MetFOSAA	C11H6F17NO4S	570	19.91	1.293974	3.921134	y = 88.766x + 0.0157	0.999	103	96	9	18

Instrumental parameters used for UPLC-HRMS

All PFASs were chromatographically separated by using a Raptor ARC-18 5 um EXP guard column (Restek, Bellefonte, PA, USA). Moreover, a CMB WR C18 50 × 4.6 mm, 10 μm (PerkinElmer Italia SPA, Milan, Italy), was introduced before the injector to allow delaying of eventual PFASs already present in the system. The mobile phase consisted of phase A (20 mM aqueous ammonium formate) and B (MeOH). The gradient started with 20% B, which reached 95% B at the 20th min and was kept in this condition for 10 min. At the 30th minute, the initial conditions (20% B) were reached and kept for 4 min for riequilibration. The run was performed at 0.3 mL min-1, with a total duration of 35 min. Regarding the detector parameters, the capillary and vaporizer temperatures were set at 330 and 280 °C, respectively, the sheath and auxiliary gas were set at 5 and 15 arbitrary units, and the electrospray voltage was set at 3.50 kV, operating in negative mode. The full scan (FS) acquisition (70,000 FWHM resolution, scan range 200–950 m/z, 1E6 of automatic gain control AGC, maximum injection time of 200 ms) was combined with a data-independent acquisition (DIA) mode for the confirmatory response, based on an inclusion list that operated at 35,000 FWHM resolution, 5E4 AGC target, maximum injection time of 100 ms, and isolation window of 2 m/z.

Table S2: Concentrations of PFASs in liver and muscle tissue and comparison between matrices obtained from roe deer from Oltrepo' Pavese, Italy. Data are reported in $\mu g \cdot kg^{-1}$, detection frequencies (concentration > LOQ) are reported in %.

				Percentile				
	**Matrix**	**Mean±SD**	**Min-Max**	**25th**	**Median**	**75th**	**DF%**	**pvalue**
**PFBA**	Meat	0.051±0.119	0-0.679	0	0	0.051	40	>0,05
	Liver	0.018±0.038	0-0.170	0	0	0.002	25	
**PFPeA**	Meat	0.026±0.032	0-0.108	0	0	0.058	42.5	>0.05
	Liver	0.015±0.027	0-0.068	0	0	0.015	25	
**PFHxA**	Meat	N.D.		0	0	0	0	
	Liver	N.D.		0	0	0	0	
**PFHpA**	Meat	0.065±0.115	0-0.302	0	0	0.047	25	>0.05
	Liver	0.079±0.148	0-0.697	0	0	0.197	35	
**PFOA**	Meat	0.069±0.101	0-0.254	0	0	0.178	40	>0.05
	Liver	0.156±0.323	0-1.844	0	0.073	0.211	70	
**PFNA**	Meat	0.082±0.142	0-0.355	0	0.002	0.051	50	<0.001
	Liver	0.366±0.277	0-1	0.146	0.373	0.550	95	
**PFDA**	Meat	0.007±0.013	0-0.032	0	0	0.007	25	>0.05
	Liver	0.006±0.012	0-0.031	0	0	0	20	
**FOUEA**	Meat	N.D.	0	0	0	0		
	Liver	N.D.	0	0	0	0		
**NADONA**	Meat	N.D.	0	0	0	0		
	Liver	N.D.	0	0	0	0		
**PFBS**	Meat	0.065±0.118	0-0.355	0	0	0.034	25	>0.05
	Liver	0.081±0.152	0-0.612	0	0	0.064	27.5	
**PFHxS**	Meat	0.06135±0.11033	0-0.318	0	0	0.035	25	>0.05
	Liver	0.09445±0.16312	0-0.5538	0	0	0.213	30	
**PFOS**	Meat	0.2961±0.751	0-8.019	0	0	0.277	47.5	<0.001
	Liver	0.666±0.635	0.114-3.941	0.351	0.526	0.782	100	
**FOSA**	Meat	0.003±0.006	0-0.019	0	0	0	22.5	<0.05
	Liver	N.D.	0	0	0	0		
**6-2FTS**	Meat	0.037±0.0704	0-0.274	0	0	0.026	42.5	>0.05
	Liver	0.033±0.071	0-0.335	0	0	0.033	47.5	
**8-2FTS**	Meat	0.043±0.063	0-0.213	0	7.05E-05	0.045	50	>0.05
	Liver	0.049±0.077	0-0.359	0	0.013	0.065	50	

Table S3: PFASs detected in liver of roe deer, comparison between urbanized and rural area. Data are reported in μg·kg^{-1}; Detection frequencies (DF%): number of samples > LOQ.

	Area	Mean ± SD	Min - Max	Percentiles			DF %	p-value
				25th	Median	75th		
**PFBA**	Urbanized	0.025 ± 0.044	0 - 0.17	0	0	0.059	15	>0.05
	Rural	0.013 ± 0.033	0 - 0.119	0	0	0	35	
**PFPeA**	Urbanized	0.022 ± 0.031	0 - 0.068	0	0	0.062	15	>0.05
	Rural	0.010 ± 0.024	0 - 0.064	0	0	0	35	
**PFHpA**	Urbanized	0.123 ± 0.183	0 - 0.697	0	0	0.237	25	>0.05
	Rural	0.038 ± 0.087	0 - 0.254	0	0	0.003	45	
**PFOA**	Urbanized	0.380 ± 0.11	0 - 0.250	0.014	0.156	0.213	60	>0.05
	Rural	0.164 ± 0.427	0 - 1.844	0	0.018	0.142	75	
**PFNA**	Urbanized	0.445 ± 0.276	0 - 0.859	0.180	0.383	0.534	100	>0.05
	Rural	0.387 ± 0.365	0.037 - 1	0.156	0.379	0.614	85	
**PFDA**	Urbanized	0.006 ± 0.012	0 - 0.031	0	0	0	20	>0.05
	Rural	0.005 ± 0.011	0 - 0.032	0	0	0	20	
**PFBS**	Urbanized	0.119 ± 0.180	0 - 0.612	0	0	0.267	20	>0.05
	Rural	0.048 ± 0.113	0 - 0.378	0	0	0	40	
**PFHxS**	Urbanized	0.128 ± 0.186	0 - 0.553	0	0	0.300	20	>0.05
	Rural	0.066 ± 0.136	0 - 0.460	0	0	0	40	
**PFOS**	Urbanized	0.786 ± 0.406	0.114 - 8.019	0.332	0.535	0.719	100	>0.05
	Rural	0.596 ± 0.839	0.264 - 3.940	0.358	0.549	0.929	100	
**6-2 FTS**	Urbanized	0.042 ± 0.064	0 - 0.291	0	0.007	0.032	45	>0.05
	Rural	0.028 ± 0.082	0 - 0.335	0	0.008	0.034	50	
**8-2 FTS**	Urbanized	0.065 ± 0.059	0 - 0.25	0	0.017	0.053	50	>0.05
	Rural	0.039 ± 0.095	0 - 0.359	0	0.035	0.083	50	

Table S4: PFASs detected in muscle of roe deer, comparison between urbanized and rural area. Data are reported in μg·kg^{-1}; Detection frequencies (DF%): number of samples > LOQ.

	Area	Mean ± SD	Min - Max	Percentiles			DF%	p-value
				25th	Median	75th		
**PFBA**	Urbanized	0.070 ± 0.153	0 - 0.679	0	0	0.091	35	> 0.05
	Rural	0.034 ± 0.071	0 - 0.277	0	0	0.031	45	
**PFPeA**	Urbanized	0.036 ± 0.036	0 - 0.108	0	0	0.065	35	> 0.05
	Rural	0.021 ± 0.027	0 - 0.068	0	0	0.044	45	
**PFHpA**	Urbanized	0.095 ± 0.133	0 - 0.302	0	0	0.253	15	> 0.05
	Rural	0.039 ± 0.092	0 - 0.278	0	0	0	35	
**PFOA**	Urbanized	0.095 ± 0.109	0 - 0.255	0	0.039	0.205	25	> 0.05
	Rural	0.048 ± 0.089	0 - 0.254	0	0	0.054	55	
**PFNA**	Urbanized	0.166 ± 0.290	0 - 0.355	0	0.004	0.334	55	> 0.05
	Rural	0.063 ± 0.129	0 - 0.356	0	8.40E-04	0.02	65	
**PFDA**	Urbanized	0.011 ± 0.014	0 - 0.031	0	0	0.029	15	> 0.05
	Rural	0.005 ± 0.011	0 - 0.032	0	0	0	35	
**PFBS**	Urbanized	0.096 ± 0.138	0 - 0.355	0	0	0.227	30	> 0.05
	Rural	0.038 ± 0.091	0 - 0.295	0	0	0.003	35	
**PFHxS**	Urbanized	0.091 ± 0.129	0 - 0.317	0	0	0.245	15	> 0.05
	Rural	0.034 ± 0.082	0 - 0.276	0	0	0	35	
**PFOS**	Urbanized	0.522 ± 1.772	0 - 8.019	0	0	0.354	50	> 0.05
	Rural	0.077 ± 0.153	0 - 0.484	0	0.003	0.03	45	
**FOSA**	Urbanized	0.005 ± 0.007	0 - 0.019	0	0	0.014	15	> 0.05
	Rural	0.002 ± 0.006	0 - 0.014	0	0	0	30	
**6-2FTS**	Urbanized	0.055 ± 0.079	0 - 0.214	0	0	0.131	40	> 0.05
	Rural	0.025 ± 0.063	0 - 0.274	0	0	0.012	45	
**8-2 FTS**	Urbanized	0.061 ± 0.074	0 - 0.213	0	0.034	0.135	50	> 0.05
	Rural	0.032 ± 0.051	0 - 0.188	0	7.04E-05	0.038	50	

Table S5: PFASs detected in liver of roe deer, comparison between sexes. Data are reported in μg·kg^{-1}; Detection frequencies (DF%): number of samples > LOQ.

	Sex	Mean ± SD	Min - Max	Percentiles			DF %	p-value
				25th	Median	75th		
**PFBA**	Female	0.037 ± 0.048	0 - 0.17	0	0.004	0.064	50	< .001
	Male	N.D.	0	0	0	0	0	
**PFPeA**	Female	0.031 ± 0.032	0 - 0.068	0	0.029	0.063	50	< .001
	Male	N.D.	0	0	0	0	0	
**PFHpA**	Female	0.123 ± 0.128	0 - 0.348	0	0.1	0.237	60	0.002
	Male	0.035 ± 0.156	0 - 0.697	0	0	0	10	
**PFOA**	Female	0.125 ± 0.111	0 - 0.299	0.006	0.169	0.214	80	0.134
	Male	0.410 ± 1.194	0 - 1844	0	0.046	0.136	55	
**PFNA**	Female	0.424 ± 0.204	0.115 - 0.854	0.328	0.417	0.553	100	0.376
	Male	0.403 ± 0.417	0 - 1.503	0.101	0.243	0.667	90	
**PFDA**	Female	0.007 ± 0.012	0 - 0.031	0	0	0	20	0.892
	Male	0.006 ± 0.012	0 - 0.032	0	0	0	20	
**PFBS**	Female	0.163 ± 0.183	0 - 0.612	0	0.118	0.291	60	< .001
	Male	N.D.	0	0	0	0	0	
**PFHxS**	Female	0.177 ± 0.194	0 - 0.553	0	0.113	0.325	50	0.003
	Male	0.011 ± 0.046	0 - 0.209	0	0	0	10	
**PFOS**	Female	0.719 ± 0.273	0.264 - 1.215	0.576	0.713	0.877	100	0.002
	Male	0.613 ± 0.865	0.114 - 3.940	0.297	0.371	0.492	100	
**6-2 FTS**	Female	0.066 ± 0.089	0 - 0.335	0.024	0.034	0.049	95	< .001
	Male	N.D.	0	0	0	0	0	
**8-2 FTS**	Female	0.099 ± 0.085	0.026 - 0.359	0.048	0.071	0.099	100	< .001
	Male	N.D.	0	0	0	0	0	

Table S6: PFASs detected in muscle of roe deer, comparison between sexes. Data are reported in μg·kg^{-1}; Detection frequencies (DF%): number of samples > LOQ.

	Sex	Mean ± SD	Min - Max	Percentiles			DF%	p-value
				25th	Median	75th		
**PFBA**	Female	0.056 ± 0.078	0 - 0.277	0	0.017	0.097	55	> 0.05
	Male	0.044 ± 0.151	0 - 0.679	0	0	0.005	25	
**PFPeA**	Female	0.046 ± 0.031	0 - 0.108	0.033	0.059	0.066	75	< .001
	Male	0.005 ± 0.013	0 - 0.044	0	0	0	10	
**PFHpA**	Female	0.130 ± 0.135	0 - 0.302	0	0.094	0.26	50	< .001
	Male	N.D.	0	0	0	0	0	
**PFOA**	Female	0.118 ± 0.116	0 - 0.254	0	0.131	0.238	55	<0.05
	Male	0.019 ± 0.046	0 - 0.197	0	0	0.009	25	
**PFNA**	Female	0.217 ± 0.286	0 - 0.121	0.004	0.175	0.342	95	< .001
	Male	0.007 ± 0.017	0 - 0.063	0	0	0.002	30	
**PFDA**	Female	0.015 ± 0.015	0 - 0.032	0	0.014	0.029	50	< .001
	Male	N.D.	0	0	0	0	0	
**PFBS**	Female	0.131 ± 0.140	0 - 0.355	0	0.064	0.256	55	< .001
	Male	N.D.	0	0	0	0	25	
**PFHxS**	Female	0.122 ± 0.131	0 - 0.317	0	0.071	0.25	50	< .001
	Male	N.D.	0	0	0	0	0	
**PFOS**	Female	0.587 ± 0.176	0 - 8.019	0.011	0.301	0.366	90	< .001
	Male	0.005 ± 0.023	0 - 0.106	0	0	0	5	
**FOSA**	Female	0.007 ± 0.008	0 - 0.019	0	0	0.014	45	< .001
	Male	N.D.	0	0	0	0	0	
**6-2FTS**	Female	0.074 ± 0.085	0 - 0.274	0.005	0.03	0.151	85	< .001
	Male	N.D.	0	0	0	0	0	
**8-2 FTS**	Female	0.086 ± 0.064	0 - 0.213	0.036	0.046	0.14	95	< .001
	Male	N.D.	0	0	0	0	0	

Table S7: PFASs detected in liver of roe deer, comparison between age classes. Data are reported in μg·kg^{-1}; detection frequencies (DF%): number of samples > LOQ.

	Age	Mean ± SD	Min - Max	Percentiles			DF%	pvalue
				25th	Median	75th		
**PFBA**	< 2 y.o.	0.027 ± 0.045	0 - 0.17	0	0	0.059	35	> 0.05
	> 2 y.o.	0.009 ± 0.029	0 - 0.119	0	0	0	15	
**PFPeA**	< 2 y.o.	0.022 ± 0.031	0 - 0.068	0	0	0.062	35	> 0.05
	> 2 y.o.	0.009 ± 0.022	0 - 0.064	0	0	0	15	
**PFHpA**	< 2 y.o.	0.088 ± 0.126	0 - 0.348	0	0	0.226	35	> 0.05
	> 2 y.o.	0.070 ± 0.169	0 - 0.697	0	0	0.008	35	
**PFOA**	< 2 y.o.	0.091 ± 0.111	0 - 0.299	0	0.016	0.209	55	> 0.05
	> 2 y.o.	0.446 ± 0.427	0 - 1.844	0.018	0.129	0.192	80	
**PFNA**	< 2 y.o.	0.305 ± 0.245	0 - 0.854	0.126	0.243	0.414	100	<0.05
	> 2 y.o.	0.528 ± 0.358	0 - 1	0.251	0.523	0.653	100	
**PFDA**	< 2 y.o.	0.007 ± 0.013	0 - 0.031	0	0	0.007	25	> 0.05
	> 2 y.o.	0.005 ± 0.011	0 - 0.032	0	0	0	15	
**PFBS**	< 2 y.o.	0.116 ± 0.178	0 - 0.612	0	0	0.259	40	> 0.05
	> 2 y.o.	0.046 ± 0.114	0 - 0.378	0	0	0	20	
**PFHxS**	< 2 y.o.	0.123 ± 0.182	0 - 0.553	0	0	0.296	35	> 0.05
	> 2 y.o.	0.065 ± 0.139	0 - 0.460	0	0	0.002	25	
**PFOS**	< 2 y.o.	0.527 ± 0.268	0.114 - 8.019	0.355	0.484	0.622	100	> 0.05
	> 2 y.o.	0.805 ± 0.846	0.152 - 3.940	0.347	0.596	0.878	100	
**6-2 FTS**	< 2 y.o.	0.029 ± 0.065	0 - 0.291	0	0	0.032	45	> 0.05
	> 2 y.o.	0.037 ± 0.077	0 - 0.335	0	0.011	0.033	50	
**8-2 FTS**	< 2 y.o.	0.040 ± 0.063	0 - 0.25	0	0.013	0.05	50	> 0.05
	> 2 y.o.	0.058 ± 0.090	0 - 0.359	0	0.024	0.082	50	

Table S8: PFASs detected in muscle of roe deer, comparison between age classes. Data are reported in µg·kg^{-1}; detection frequencies (DF%): number of samples > LOQ.

	Age Class	Mean ± SD	Minimo	Percentiles 25th	Median	75th	DF%	pvalue
**PFBA**	< 2 y.o.	0.035 ± 0.057	0 - 0.197	0	0	0.058	40	> 0.05
	> 2 y.o.	0.065 ± 0.159	0 - 0.679	0	0	0.041	40	
**PFPeA**	< 2 y.o.	0.034 ± 0.034	0 - 0.108	0	0.044	0.065	55	> 0.05
	> 2 y.o.	0.016 ± 0.026	0 - 0.069	0	0	0.044	30	
**PFHpA**	< 2 y.o.	0.090 ± 0.127	0 - 0.302	0	0	0.248	35	> 0.05
	> 2 y.o.	0.041 ± 0.099	0 - 0.288	0	0	0	15	
**PFOA**	< 2 y.o.	0.079 ± 0.113	0 - 0.254	0	0	0.211	35	> 0.05
	> 2 y.o.	0.058 ± 0.088	0 - 0.254	0	0	0.076	45	
**PFNA**	< 2 y.o.	0.164 ± 0.292	0 - 0.337	0	1.00E-03	0.337	50	> 0.05
	> 2 y.o.	0.059 ± 0.121	0 - 0.355	6.30E-04	0.003	0.028	75	
**PFDA**	< 2 y.o.	0.011 ± 0.015	0 - 0.033	0	0	0.029	35	> 0.05
	> 2 y.o.	0.004 ± 0.011	0 - 0.029	0	0	0	15	
**PFBS**	< 2 y.o.	0.084 ± 0.123	0 - 0.355	0	0	0.218	40	> 0.05
	> 2 y.o.	0.046 ± 0.113	0 - 0.338	0	0	1.00E-03	2.50E+01	
**PFHxS**	< 2 y.o.	0.080 ± 0.115	0 - 0.296	0	0	0.204	35	> 0.05
	> 2 y.o.	0.042 ± 0.104	0 - 0.317	0	0	0	15	
**PFOS**	< 2 y.o.	0.529 ± 0.177	0 - 8.019	0	0.002	0.353	50	> 0.05
	> 2 y.o.	0.063 ± 0.130	0 - 0.376	0	0	0.024	45	
**FOSA**	< 2 y.o.	0.004 ± 0.006	0 - 0.016	0	0	0.012	30	> 0.05
	> 2 y.o.	0.002 ± 0.006	0 - 0.019	0	0	0	15	
**6-2FTS**	< 2 y.o.	0.022 ± 0.054	0 - 0.182	0	0	0.006	40	> 0.05
	> 2 y.o.	0.051 ± 0.082	0 - 0.274	0	0	0.066	45	
**8-2 FTS**	< 2 y.o.	0.033 ± 0.055	0 - 0.213	0	0	0.037	45	> 0.05
	> 2 y.o.	0.053 ± 0.069	0 - 0.188	0	0.018	0.126	55	
